# Cutoff lensing: predicting catalytic sites in enzymes

**DOI:** 10.1038/srep14874

**Published:** 2015-10-08

**Authors:** Simon Aubailly, Francesco Piazza

**Affiliations:** 1 Université d’Orléans, Centre de Biophysique Moléculaire, CNRS-UPR4301, Rue C. Sadron, 45071, Orléans, France

## Abstract

Predicting function-related amino acids in proteins with unknown function or unknown allosteric binding sites in drug-targeted proteins is a task of paramount importance in molecular biomedicine. In this paper we introduce a simple, light and computationally inexpensive structure-based method to identify catalytic sites in enzymes. Our method, termed *cutoff lensing*, is a general procedure consisting in letting the cutoff used to build an elastic network model increase to large values. A validation of our method against a large database of annotated enzymes shows that optimal values of the cutoff exist such that three different structure-based indicators allow one to recover a maximum of the known catalytic sites. Interestingly, we find that the larger the structures the greater the predictive power afforded by our method. Possible ways to combine the three indicators into a single figure of merit and into a specific sequential analysis are suggested and discussed with reference to the classic case of HIV-protease. Our method could be used as a complement to other sequence- and/or structure-based methods to narrow the results of large-scale screenings.

With the rapid development and refinement of experimental techniques for protein structure determination at high resolution, predicting functional sites is a major issue in modern molecular biology in many protein families^1^,^2^,^3^,^4^,^5^,^6^,^7^.

The swiftly growing amount of structural and sequence data poses big challenges and offers great opportunities to test automated prediction algorithms and platforms. Several approaches have been used to identify critical function-related sites (sometimes referred to as *hotspots*) in proteins. Most of these methods imply structural and/or sequence conservation information^8^,^9^,^10^,^11^,^12^,^13^,^14^.

Purely sequence conservation approaches use phylogenetic information, relying on the idea that functional sites are conserved during evolution. Typically, such algorithms proceed through the alignment of a great number of different sequences and the ensuing computation of different conservation scores^7^,^15^,^16^,^17^,^18^. Other approaches can be found in the literature, typically combining sequence-related information with structural data to achieve higher prediction rates^19^,^20^,^21^.

Among the structure-based algorithms developed to identify and predict function-related sites in proteins, an appealing and promising class is that of coarse-grained (CG)^22^,^23^ approaches based on elastic-network models (ENM)^24^,^25^,^26^,^27^,^28^,^29^. The ENM^30^ and its CG versions^31^,^32^ are light and computationally inexpensive tools that have proved tremendously effective in dissecting function-related vibrational patterns in proteins, both embodied in low-frequency collective normal modes^33^,^34^,^35^,^36^,^37^ and, more subtly, related to high-frequency localized vibrations^28^,^38^,^39^,^40^,^41^.

Often, graph-theoretical tools have been employed in combination with ENM-related approaches^42^,^43^,^44^,^45^,^46^,^47^,^48^,^49^,^50^ to identify hotspots and binding interfaces. In these methods, a protein structure is mapped onto a network by means of some rule. In one simple CG example, nodes may represent amino acids while edges embody pair-wise interactions that can be obtained either from the study of equilibrium structures^47^,^48^ or from molecular dynamics (MD) simulations^51^. Typical graph-theoretical measures employed for such analyses include connectivity^24^,^48^, different measures of centrality^16^,^45^,^47^,^52^,^53^,^54^, betweenness and cluster coefficient^16^.

It is clear from the above discussion that a successful strategy to predict functional sites in proteins has to rely on a composite approach, combining information from sequence conservation with structure-based analyses. In turn, the latter should combine different indicators, related to the physical-chemical properties of amino acid environments and to patterns of chemical and topological connectivity.

In this paper we focus on the prediction of catalytic sites in enzymes based on an original ENM-based strategy. Atomistic approaches devised to identify residues involved in catalysis in enzymes are not new^55^. More recently, approaches specifically relying on sophisticated electrostatic calculations have been introduced^56^,^57^. Conversely, coarse-grained models have been relatively less exploited to solve this specific problem^29^,^58^,^59^. Yet, ENM-based tools are light (they can be applied to large databases of structures) and can be readily extended to perform all-residue searches in many structures. Moreover, CG topology-based methods have the advantage to strip the structure of most chemical details so as to bring to the surface purely topological features. This appears particularly important in the case of enzymes, as often sites that are involved in the catalytic action are intriguingly found far from the annotated catalytic sites^60^,^61^.

Our method combines three different indicators, two graph-theoretical measures with an original scale of local *stiffness* in a method that we termed *cutoff lensing*. The main idea is that catalytic sites can be spotlighted by employing elastic network models whose connectivity is increased beyond currently employed values. In ENMs, a spring is stretched between all pairs of residues that are separated in the equilibrium structure by a distance less than a specified cutoff length *R*_*c*_. Typically employed values for protein models coarse-grained at the level of amino acids vary in the 10–13 Å range^32^,^62^,^63^,^64^,^65^, even if values greater than 13 Å have also been considered episodically^66^,^67^,^68^. In principle, larger values of the cutoff are unphysical, as the connectivity graph becomes nearly fully connected as *R*_*c*_ attains a value comparable with the protein size. Nevertheless, we have found that specific, function-related sites can be singled out in such regimes by using indicators associated with topological and structural measures of connectedness and stiffness. Remarkably, a scan of increasing values of the cutoff shows that there exists an optimum range where our structural indicators are the most sensitive in detecting catalytic sites known from experiments. This *lensing* effect can thus be used to predict the location of functional sites in unannotated proteins.

The paper is organized as follows. In the next section we provide the description of the cutoff lensing method and introduce three structure-based indicators. In the following section we check the predictive power of our indicators against the pool of annotated catalytic sites in a large database of enzymes. Finally, we discuss our results and provide a working summary of our method.

## Methods

We model a given protein consisting of *N* amino acids as an ensemble of *N* fictitious particles occupying the equilibrium positions of the corresponding *α*-carbons, as found in the experimental structure. All particles have the same mass *M*, which we set equal to the average amino acid mass, *M* = 120 Da (as the fictitious particles occupy the equilibrium positions of amino acids, *i.e.* are located on the corresponding *α*-carbons, we will use the words particles and (amino acid) residues interchangeably). Each particle interacts with its neighbours, as specified by the cutoff distance *R*_*c*_. More precisely, residues *i* and *j* interact if |***R***_*i*_ − ***R***_*j*_| ≤ *R*_*c*_, where ***R***_*i*_ denotes the position vector of the *i*-th residue in the equilibrium structure. Let ***r***_*i*_ denote the instantaneous position vector of the *i*-th residue. The system potential energy reads


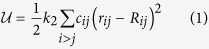


where *r*_*ij*_ = |***r***_*ij*_| = |***r***_*i*_ − ***r***_*j*_|, *R*_*ij*_ = |***R***_*ij*_| = |***R***_*i*_ − ***R***_*j*_| are the inter-particle instantaneous and equilibrium distances, respectively. Interacting pairs are specified through the contact matrix


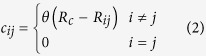


*θ*(*x*) denoting the Heaviside step function. Eq. (1) amounts to building a network of Hookean springs joining pairs of residues separated by a distance smaller than *R*_*c*_. Normal modes (NM) are computed by diagonalizing the (mass-weighted) Hessian matrix,


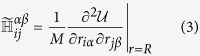


which gives 3*N* − 6 normal modes 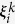, *k* = 7, 8, …, 3*N* with non-zero eigenvalues 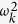. Here, greek indexes indicate Cartesian spatial directions. It is straightforward to show from Eq. (1) that


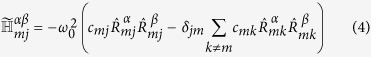


where *δ*_*jm*_ is the Kronecker symbol, 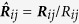 and 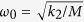. Following previous studies, we fix *k*_2_ = 5 kcal/mol/Å^2^ 63.

### Constructing structural hotspot indicators

The basic idea of our method rests on the evidence reported by several studies that hotspot/functional sites in proteins are generally found in stiff/rigid regions^59^,^69^,^70^,^71^. Analogously, it has been shown that functional residues tend to move independently from the rest of the structure, involving high-frequency localized vibrations (the stiffer the bonds, the higher the frequency)^62^,^63^,^72^,^73^.

Structural rigidity can be gauged by many indicators, that assess the different *flavours* associated with it. The simplest and more intuitive method, albeit unsuitable for automated screening of large structure databases, would be to measure fluctuations directly via MD simulations, such as in ref. 74. Alternatively, but more indirectly, rigidity can be related to the local number of neighbours in the protein connectivity graph. A series of recent studies has demonstrated a rather surprising agreement between the location of catalytic sites in enzymes and the localization patterns of nonlinear vibrational modes known as discrete breathers (DB)^38^,^39^. Such observations have been rationalized in terms of a *spectral measure of local stiffness*, based on the localization properties of high-frequency normal modes^62^.

Here we introduce an original method based on a blend of suitable structural indicators combined with *cutoff lensing*, *i.e.* an analysis where the cutoff *R*_*c*_ is let increase beyond physically realistic values. The key feature of this method is a selective sharpening of the predictive power of our indicators at specific intermediate values of *R*_*c*_.

A *spectral* stiffness measure can be computed by looking at the contribution of a reduced set of high-frequency NMs, 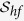, to atomic fluctuations, that is


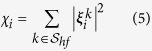


In the following we shall consider the last five high-frequency NMs, *i.e.* 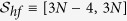. The rationale behind Eq. (5) comes from the observation that fast normal modes tend to be localized at hotspot sites^62^, *i.e.* sites that act as efficient energy storage and accumulation centers, typically flagging highly connected and buried regions. Along the same lines, fast modes have also been demonstrated to identify stability cores of proteins^41^, adding to the meaningfulness of definition (5). Typically, in residue-based coarse-grained ENMs the last high-frequency NMs are localized around one, two sites at most. If one considers an average number of catalytic sites per enzyme around 5 (it is 2.5 in the Catalytic Site Atlas (CSA)^75^), it appears that the minimum number of high-frequency NMs to include in the definition (5) is five (adding a few more NMs does not change appreciably our results. Adding more results in useless blurred patterns).

Following a similar rationale, we shall also consider indicators referring to the connectivity graph, notably the local connectivity,


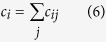


and, as already done by other authors^16^,^45^,^47^,^52^,^53^,^54^, the closeness centrality, defined as


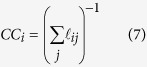


where 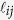 is the shortest path (in units of edge number) between nodes *i* and *j* over the connectivity graph.

The three above-defined indicators can be regarded as supplying different measures of *stiffness*. While *χ*_*i*_ gauges the *vibrational* stiffness of a given residue, *i.e.* its propensity to vibrate at high frequency with a space-localized pattern, *c*_*i*_ and *CC*_*i*_ exquisitely quantify the *topological* stiffness, in the sense of number of outgoing bonds (*c*_*i*_) or shortest paths between two given locations flowing through *i* (*CC*_*i*_).

**Figure 1:**
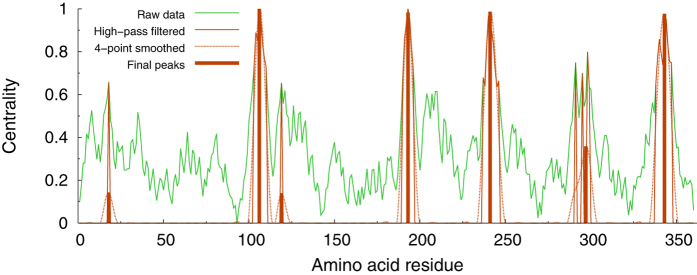
Illustration of the computation of the reduced closeness centrality indicator through the different sequential steps described in the text. The patterns are normalized to the maximum value occurring in the sequence. The final peaks flag the potentially functional sites. The calculations refer to Arginin Glycineaminotransferase (PDB code 1JDW).

As a general rule, the raw measures of *χ*_*i*_, *c*_*i*_ and *CC*_*i*_ result in rather rugged and irregular patterns with many peaks and troughs for a given protein sequence. Our goal is to extract from such patterns the most relevant peaks as flags for potentially functional sites. To this aim, we apply a high-pass *filtering* procedure, by keeping for a given indicator pattern only the values above a specified number of standard deviations (computed over the whole sequence). More precisely,


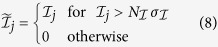


where 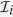 is the considered indicator, 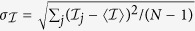 its standard deviation and 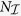 an indicator-dependent high-pass threshold. In our analysis, we fixed 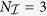 for 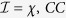 and 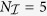 for 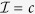. Our final site predictions are then obtained as the locations flagged by the peaks that survive in the pattern after the high-pass filtering. In the case of *CC*, the patterns showed overly rugged profiles (see Fig. 1), which resulted in a large number of close, quasi-degenerate peaks after the high-pass filtering. Accordingly, in order to eliminate the degeneracy associated with multiple-peak structures, we applied a 4-point smoothing procedure^76^ to the filtered patterns, so as to automatically make the excessively degenerate structures coalesce in one single-peak prediction. The whole procedure is illustrated in Fig. 1.

## Results: the cutoff lensing effect

**Figure 2:**
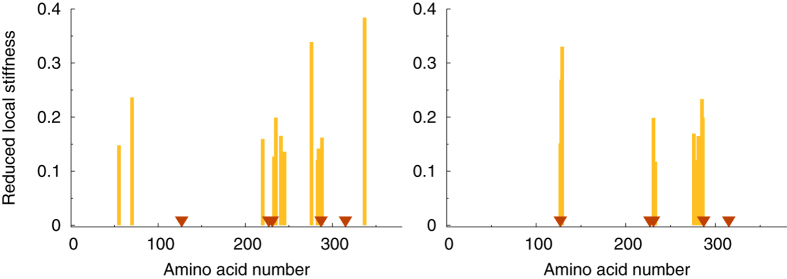
Illustration of the *cutoff lensing* effect. Plot of the reduced stiffness pattern 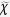, Eq. (8), for Arginin Kinase (PDB code 1BG0). Cutoff *R*_*c*_ = 10 Å (left) and *R*_*c*_ = 20 Å (right). The known catalytic sites are indicated by dark triangles. Note the disappearance of some *irrelevant* peaks and the appearance of a peak at one of the catalytic sites in going from *R*_*c*_ = 10 Å to *R*_*c*_ = 20 Å.

Our idea is to inspect reduced (filtered) patterns of local spectral and topological stiffnesses in search for hot spots. One of such patterns is reported in Fig. 2 for two values of the cutoff parameter *R*_*c*_ used to construct the elastic network (see again Eq. (2)). Interestingly, one may easily remark that there is a correspondence between the location of known catalytic sites and stiffness peaks. This finding agrees with observations made by other authors along the same lines^29^,^59^. However, if we now repeat the same analysis with a higher (even if less physical value of *R*_*c*_), the surprising consequence is that the reduced pattern is sharpened down to a handful of peaks, which seem to much better pinpoint the known functional sites. Note that the observed sharpening of the predictive power implies both the *evaporation* and the *relocation* of some peaks. We term this effect altogether *cutoff lensing*.

The logical questions to ask in view of such findings are (i) whether these effects also characterize the other indicators and (ii) whether there exists an optimum value of *R*_*c*_, corresponding to the maximum overlap between (generalized) stiffness peaks and catalytic sites, beyond which the patterns get blurred again and one correspondingly loses predictive power. The latter possibility, in particular, seems highly realistic, as one expects sites to be no longer distinguishable (with respect to whatever measure) in nearly fully connected networks.

**Figure 3:**
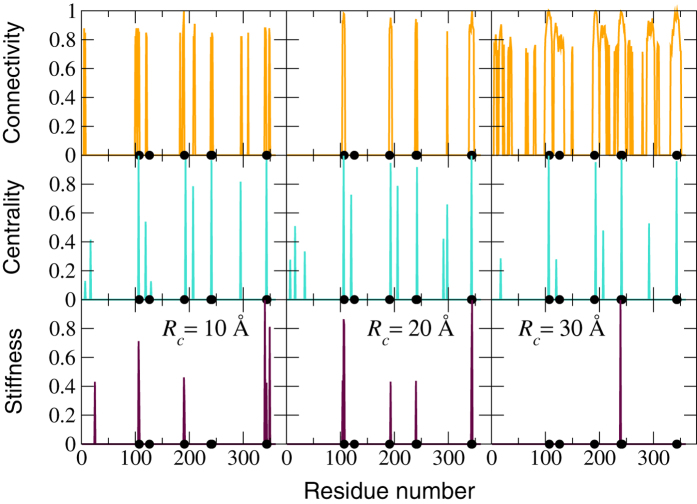
Reduced and normalized connectivity, closeness centrality and stiffness patterns computed according to the prescription (8) for Arginin Glycineaminotransferase (PDB code 1JDW) for different values of the cutoff *R*_*C*_. The annotated catalytic sites are indicated by black filled circles.

The results reported in Fig. 3 for a given enzyme seem to reply to the first question in the affirmative: intermediate values of the cutoff appear to be associated with increased predictive power. The reduced connectivity 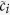 and spectral stiffness 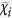 profiles suggest that intermediate values of *R*_*c*_ yield a better match between the peaks of the indicator patterns and the annotated sites. The centrality, on the contrary, provides a good match but seems at the same time rather insensitive to changes in the cutoff. It is important to observe that the number of peaks *N*_*p*_ is not constant as a function of *R*_*c*_. Of course, this information has to be included in the picture if we want to provide a statistical assessment of the predictive power of our indicators as a function of *R*_*c*_. On the one hand, *N*_*p*_ is expected to increase at high values of *R*_*c*_ for the connectivity and centrality measures, while it seems that stiffness patterns display less and less peaks as the cutoff is made larger.

**Figure 4:**
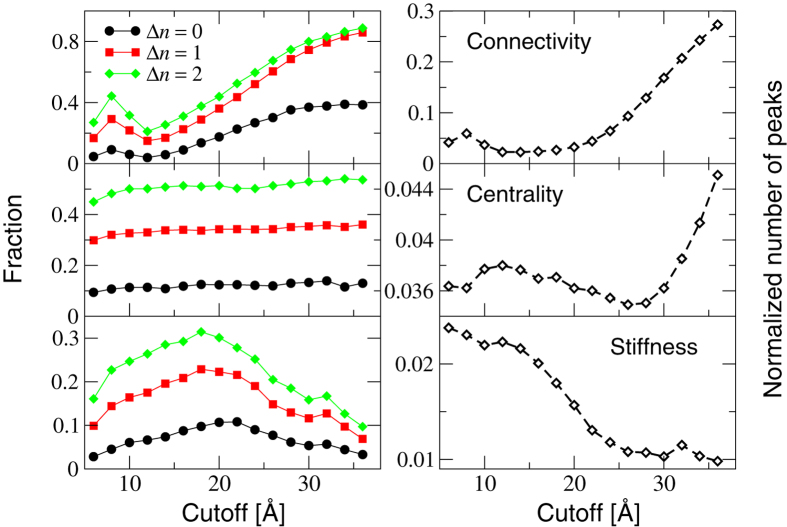
Left panels: fraction of catalytic residues within Δ*n* sites from the nearest peak versus cutoff, as computed over the ensemble of enzymes from the CSA. Right panels: average peak fraction (number of peaks divided by number of residues) computed over the whole database versus cutoff.

In order to shed further light on the above-described findings and proceed to a statistical assessment of the ability of our indicators to spotlight function-related sites, we have analyzed a pool of 835 enzyme structures from the Catalytic Site Atlas^75^. The CSA is a major resource in the field of structural biology, and provides up-to-date catalytic residue annotation for enzymes in the Protein Data Bank based on experimental structural data. The results of our ensemble analysis are reported in Fig. 4. For each indicator, we have calculated the fraction of catalytic sites that are found within a prescribed distance Δ*n* (in units of residues) along the sequence from a peak. For example, the curves at Δ*n* = 0 indicate the fraction of catalytic sites that coincide with a peak for a given indicator.

A number of interesting observations can be made by inspecting Fig. 4. The reduced connectivity 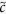 increases its predictive power at increasing values of the cutoff. However, this is a trivial consequence of the fact the number of peaks also increases as the systems become more and more connected (top right panel). Therefore, the connectivity does not appear to provide a particularly insightful spotlighting tool. On the contrary, the reduced centrality 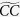 provides a comparatively more sensitive detection tool, with up to half of the whole pool of catalytic sites found at a separation of at most one residue (along the sequence) from a 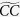 peak. Furthermore, it is seen that the predictive power of this indicator is almost insensitive to the number of peaks, which increases of course as the structures become more and more connected (middle right panel). Interestingly, the average number of peaks in the *CC* patterns displays a minimum (around *R*_*c*_ = 28 Å), which suggests that at this value of the cutoff the *reliability* of the observed predictive power of centrality is maximum.

**Figure 5:**
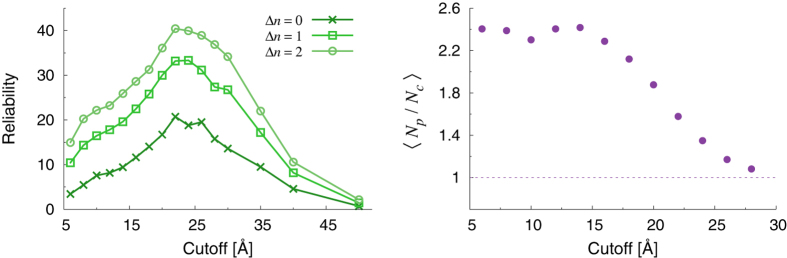
Left panel: reliability of the predictive power of reduced stiffness patterns as a function of the cutoff *R*_*C*_ (arbitrary units). The reliability is defined as the fraction of predicted catalytic sites (within Δ*n* amino acids along the sequence) divided by the fraction of stiffness peaks (number of peaks per amino acid). Right panel: Average number of peaks in the reduced stiffness patterns per catalytic site.

Of the three indicators, the reduced stiffness 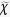 displays the most interesting behavior. The fraction of predicted sites shows a maximum at intermediate cutoff values (around 20 Å), with up to 30% of the known catalytic sites recovered at a distance of one amino acid from a peak of reduced stiffness. Interestingly, the number of such peaks decreases towards a nearly constant value as the cutoff is increased. Most remarkably, the maximum of predictive power clearly falls in a regime where the number of peaks has attained its minimum asymptotic value, which means that the statistical significance of the prediction at the maximum is also maximum. To make this observation more quantitative, one may introduce an intuitive measure of *reliability* defined as the fraction of predicted sites divided by the number of peaks found at each value of the cutoff. This is illustrated in Fig. 5 (left panel). It is clear that the predictions made from the reduced stiffness patterns correspond to a maximum of reliability at the intermediate cutoff *R*_*c*_ ≃ 22 Å. This suggests that the cutoff lensing effect can be effectively employed to predict the location of catalytic sites or to substantiate the predictions made by means of other methods based on different arguments. This is also confirmed by the observation that the highest number of predicted sites and maximum reliability corresponds to roughly one stiffness peak per catalytic site (see right panel in Fig. 5). This suggests that the optimality condition of maximum predictive power is achieved with the least number of unassociated peaks, *i.e.* under conditions of highly reduced redundancy.

**Figure 6:**
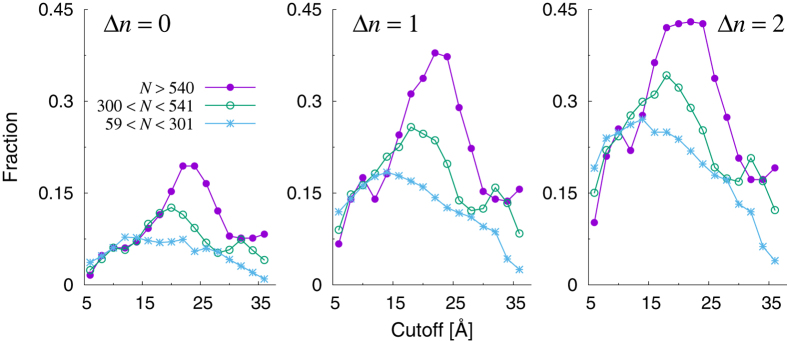
Fraction of catalytic sites within Δ*n* sites from the nearest peak of the stiffness reduced patterns computed over three different size classes in the CSA database.

It is interesting to note that the cutoff value associated with the maximum in the fraction of sites predicted by the reduced stiffness patterns increases with the size of the protein, and so does the fraction itself at the maximum. This is illustrated in Fig. 6, where we show the results of our computations performed by grouping the enzymes in three different size classes. It is clear that our algorithm is much more effective for proteins of large sizes. This remarkable finding is not restricted to stiffness patterns. In general, the fraction of catalytic sites recovered by reduced closeness and connectivity profiles is greater for enzymes of larger sizes (see supplementary material).

## Discussion, Conclusions and Perspectives

In this paper we have investigated the ability of different structure-related indicators to pinpoint the location of known catalytic sites in a large number of enzyme structures in the framework of the elastic network model. More precisely, we defined reduced *peak patterns* of (i) local connectivity, (ii) closeness centrality and (iii) structural stiffness, where the peaks retained along the protein sequence are assumed to flag potentially interesting sites. Our method is general and computationally inexpensive (see supplementary material for a benchmark test).

Our analysis shows that all three considered indicators display a considerable predictive power (up to 50% of the catalytic sites recovered within a distance of two amino acids along the sequence), when the computed peak structures are compared with the location of annotated catalytic sites in a large database of enzymes (the Catalytic Site Atlas^75^). This suggests that the three indicators can be employed in some suitable combination/sequence to make predictions in unannotated enzyme structures.

In order to find the optimal procedure to combine the three indicators, we have investigated their behavior as a function of the cutoff *R*_*c*_ used to construct the elastic networks, while monitoring in parallel the number of peaks per amino acid present in the indicator patterns. We have termed this procedure *cutoff lensing*. This analysis has revealed that optimal values of the cutoff exist in all cases. For connectivity, the fraction of known catalytic sites recovered trivially (and uninformatively) increases with the cutoff, as the number of high-connectivity peaks retained also increases. For this reason, we argue that the optimal cutoff corresponds to the highest predictive power corresponding to the least number of peaks per amino acid (about 40% of the catalytic sites recovered within a distance of two amino acids along the sequence), which means *R*_*c*_ ≈ 20 Å. By contrast, somewhat surprisingly, centrality patterns display nearly cutoff-invariant predictive power. However, the specific number of peaks displays a minimum around *R*_*c*_ = 28 Å. Therefore, we conclude that *R*_*c*_ = 28 Å can be taken as the optimality condition, reflecting the idea that for equal fractions of recovered catalytic sites the most reliable prediction is the one made with the least number of peaks.

The study of reduced stiffness patterns has led us to uncover an interesting effect, that we termed *cutoff lensing*: when the cutoff is increased, the fraction of catalytic sites spotlighted by the stiffness peak patterns displays a maximum at around *R*_*c*_ = 20 Å. Remarkably, this is achieved with a minimum degree of redundancy, as the number of peaks in the patterns (pointing to potentially interesting sites) is a minimum for *R*_*c*_ > 22 Å, while at the same time the average number of peaks per catalytic sites is about 1 on average in this range of cutoff values. We conclude that *R*_*c*_ ≃ 22 Å is the value of choice for predictions of catalytic sites made through stiffness patterns.

Remarkably, we found that the fraction of catalytic sites recovered by our indicators at the optimal cutoff is larger the larger the protein (see again Fig. 6 and also the Supplementary Material). Connectivity patterns are an exception, as at the optimal cutoff the fraction of catalytic sites recovered is nearly the same independently of the size of the enzymes.

### A sequential computation of optimal indicators to make predictions, combined scores to assess their confidence level

It is interesting to inquire whether it is possible to combine the three indicators computed at their individually optimal cutoff values in some *globally optimal* manner and what would be the meaning of such combination. The simplest operation to do is to add up the information carried by the three figures of merit, weighted by the number of peaks displayed by the corresponding patterns. This leads to introducing the following global score,


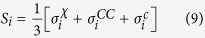


where *i* denotes the amino acid site and *σ*_*i*_ are renormalized indicator patterns, where each peak has the same height 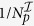 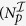 is the number of peaks in the pattern of indicator 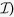, so that 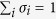.

By construction, one has 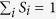. The meaning of *S*_*i*_ is to gauge the local prediction by counting the number of peaks present in the three indicator patterns, for the sake of simplicity each of them counted with an equal weight of 1/3. Within each profile, each peak is assigned an *internal* weight inversely proportional to the overall number of peaks. Again, the idea is that the larger the number of peaks, the easiest is to make a prediction at some site and consequently the less significative the prediction itself. Furthermore, the site scores *S*_*i*_ can be combined to produce an overall score *S*_Δ*n*_ for a given enzyme by adding up all the scores within Δ*n* sites from the known *N*_*c*_ catalytic sites, that is,


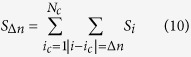


If *S*_Δ*n*_ > 0 for a given structure, our algorithm is able to provide at least one prediction. An analysis performed over the whole CSA database shows that the fraction of structures where the combined algorithm returns a prediction is 0.61 for Δ*n* = 1 and 0.68 for Δ*n* = 2 (see also Supplementary Material).

**Figure 7:**
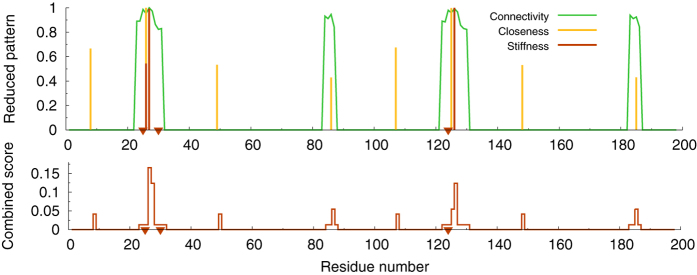
Analysis of HIV-1 protease (PDB 1A30). The upper plot shows the three reduced indicator patterns. The bottom panel illustrates the combined site score given by eq. (9).

In order to elucidate the meaning of the site scores *S*_*i*_ and global score *S*_Δ*n*_, it proves useful to concentrate on a specific enzyme. In Fig. 7, we consider the classic case of HIV-1 protease. Let us first concentrate on the profile of the combined score (9). Two facts are immediately apparent: (i) the catalytic sites appear to be all captured but (ii) there are a number of *orphan* peaks. The global scores for this enzyme are *S*_Δ*n*=1_ = 0.27 and *S*_Δ*n*=2_ = 0.29. Thus, despite the algorithm flags correctly all the catalytic sites, it does so with some degree of over-prediction (incidentally, we observe that the orphan peaks shown in the combined score profile in Fig. 7 might as well spotlight some hitherto unknown functional sites of HIV-1 protease). Of course, when applying the algorithm to unannotated structures, one does not know *a priori* which peak in the combined score is more likely to point to a catalytic site. This shows the limitations of using *only* a combined score. Analogous conclusions can be drawn by looking at the fraction of catalytic sites predicted by one or more indicators (see supplementary material). For example, closeness and stiffness reduced patterns predict 52% and 28%, respectively, of the catalytic sites within a range Δ*n* = 2. However only a fraction of 22% is predicted by both. Our conclusion is that each indicator has its specific predictive power, which should be exploited independently, while combined scores should be checked to gauge the *confidence* associated with multiple-indicator predictions.

Looking again at the example of HIV protease will make our point more clear (Fig. 7). It is not difficult to realize that a *sequential* inspection of the three separate indicator profiles at their respective optimal cutoff values is more likely to point to the known catalytic sites first. By inference, we propose that the same inspection sequence be adopted for hitherto unannotated proteins. The connectivity profiles should be examined first. These are the ones with the largest number of peaks, often coalescing to highlight extended regions. The search should be subsequently narrowed down with the corresponding closeness profile, typically featuring more localized peaks, albeit many of them likely to be orphan ones. The prediction should then be refined through the reduced stiffness patterns, the ones with the least number of peaks. Of course, extra information coming from other structure- and/or sequence-based algorithms should be used at each step in conjunction with our algorithm, if possible, to single out interesting sites.

As a final observation, we note that our choice to attribute an equal weight to the three indicators in constructing the combined score *S*_*i*_ in eq. (9) is arbitrary. It would be interesting to inquire whether there exists an optimal combination of weights 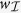 defining better generalized scores, namely 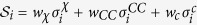 with 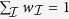. For example, one may imagine to use standard optimization techniques^77^,^78^ or genetic algorithms^79^ to efficiently determine an optimal set of weights, by training our algorithm on the CSA and other databases.

## Additional Information

**How to cite this article**: Aubailly, S. and Piazza, F. Cutoff lensing: predicting catalytic sites in enzymes. *Sci. Rep.* **5**, 14874; doi: 10.1038/srep14874 (2015).

## Supplementary Material

Supplementary Information
