# Supplementary material for: Cutoff lensing: predicting catalytic sites in enzymes
Source: Sci Rep. 2015 Oct 8;5:14874. doi: 10.1038/srep14874 (PMC4597221; doi:10.1038/srep14874)
Supplement: Supplementary Information [file srep14874-s1.pdf]

Supplementary material for the paper: Cutoff lensing: predicting catalytic sites in enzymes

Simon Aubailly[1], **Francesco Piazza**[1]

[1]Université d'Orléans, Centre de Biophysique Moléculaire, CNRS-UPR4301, Rue C. Sadron, 45071, Orléans, France

E-mail: `Francesco.Piazza@cnrs-orleans.fr`

Contents

1 Analysis of predictions by size classes **2**

2 Combining indicator patterns: global score measure **3**

3 Analysis of CPU time required to build indicator patterns **4**

1. Analysis of predictions by size classes

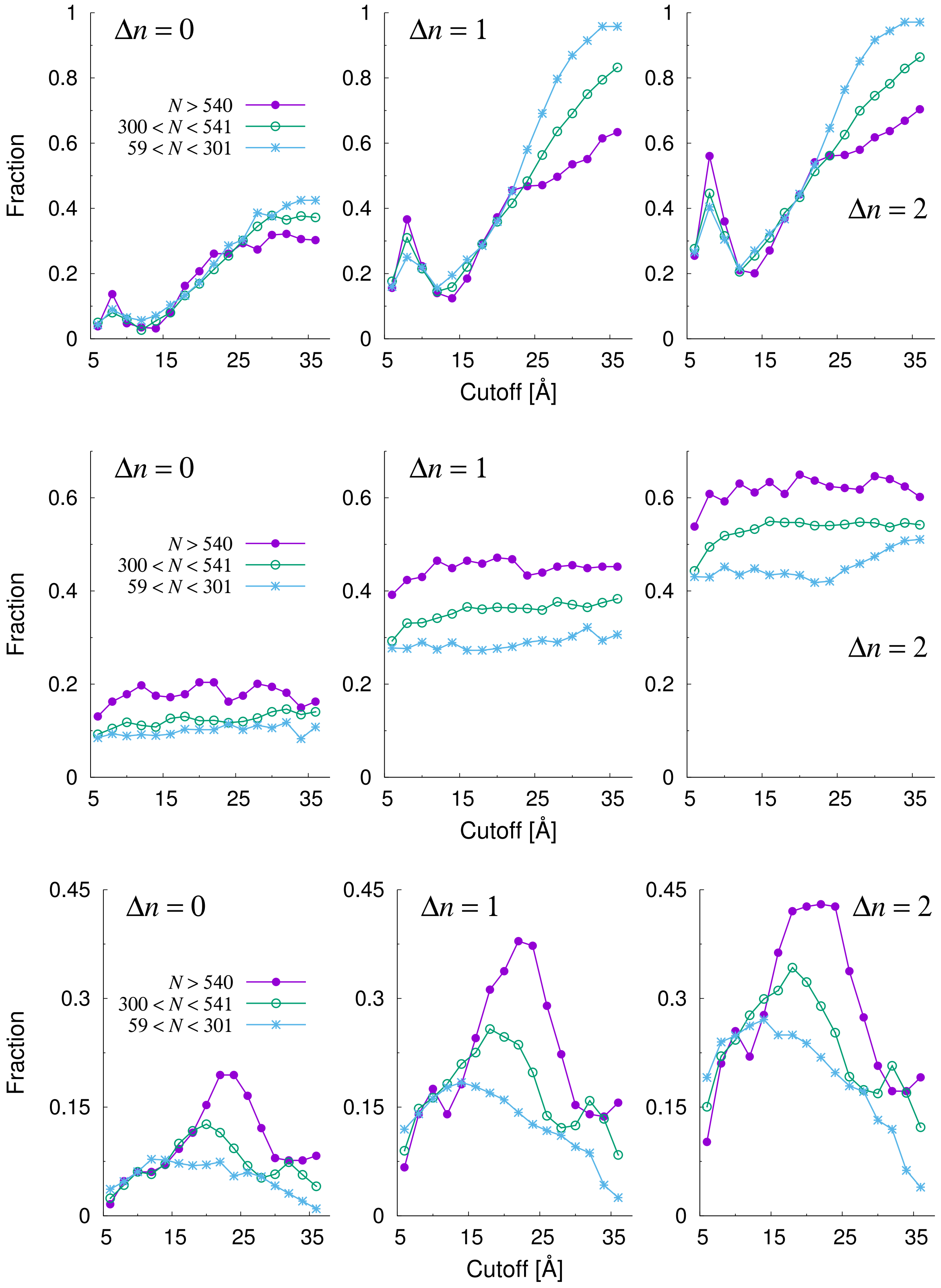

Figure 1. (Color online) fraction of catalytic sites within Δn sites from the nearest peak of the three reduced patterns computed over three different size classes in the CSA database versus cutoff. Connectivity (top), closeness (middle) and stiffness (bottom).

2. Combining indicator patterns: global score measure

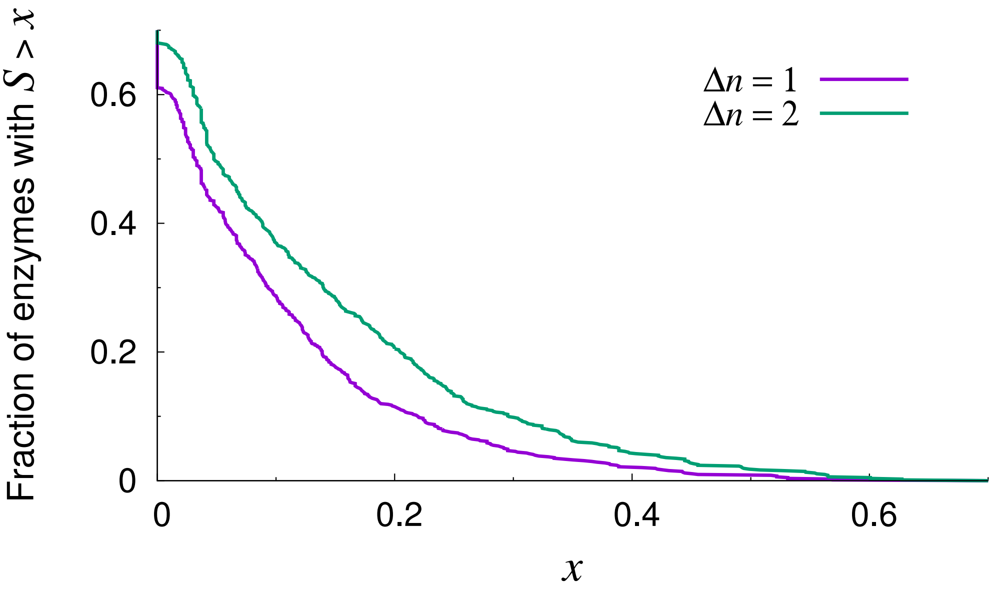

Figure 2. (Color online) Complementary cumulative distributions of global enzyme scores computed over the whole CSA database through eq. (10) in the main text. A positive score signals that a prediction has been made. The actual value of the score is a measure of the relative number of orphan peaks (*putative false positives*). As a general rule, the larger the score, the less in number and/or the smallest in height were the orphan peaks (see main text).

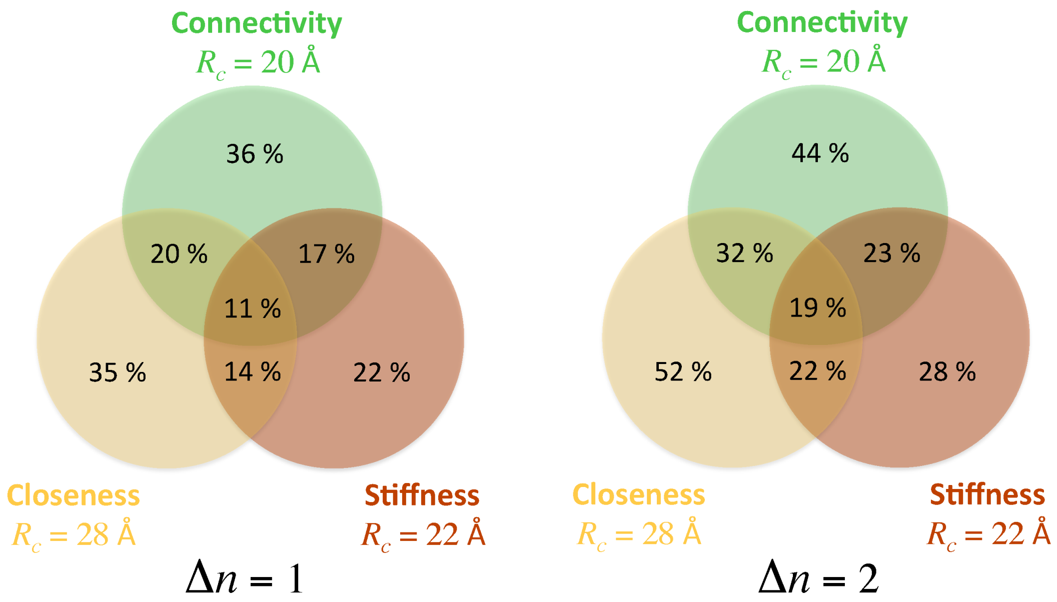

Figure 3. (Color online) Synoptic representation of the fraction of predicted catalytic sites over the CSA database at the individual optimal cutoff values for $\Delta n = 1$ and $\Delta n = 2$.

3. Analysis of CPU time required to build indicator patterns

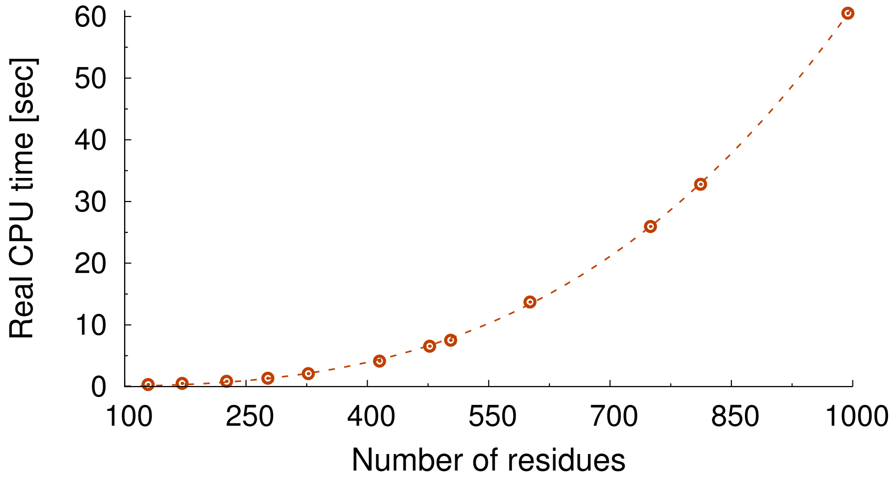

Figure 4. (Color online) CPU time required to compute reduced patterns for the three indicators illustrated in the text as a function of the number of amino acids in the enzymes. The computation refer to an ordinary desktop workstation equipped with an Intel(R) Xeon(R) CPU E5-1620 at 3.60 GHz. The dashed line is a fit with a cubic polynomial, $t = (N/N_0)^3$, which gives $N_0 = 253.2$. As expected, the overall time is dominated by the time needed to diagonalize the Hessian matrix (operation which scales as the cube of the matrix dimension).
